# Detection of Road Risk Sources Based on Multi-Scale Lightweight Networks

**DOI:** 10.3390/s24175577

**Published:** 2024-08-28

**Authors:** Rong Pang, Jiacheng Ning, Yan Yang, Peng Zhang, Jilong Wang, Jingxiao Liu

**Affiliations:** 1 School of Computing and Artificial Intelligence, Southwest Jiaotong University, Chengdu 611756, China; rongpang@my.swjtu.edu.cn; 2 China Merchants Chongqing Road Engineering Inspection Center Co., Ltd., Chongqing 400067, Chinawangjilong@cmhk.com (J.W.);; 3 National Mountain Highway Engineering Technology Research Center, Chongqing 400067, China; 4 Chongqing Transportation Planning and Technology Development Center, Chongqing 400067, China

**Keywords:** object detection, multi-scale, MobileNetV2, coordinate transformation

## Abstract

Timely discovery and disposal of road risk sources constitute the cornerstone of road operation safety. Presently, the detection of road risk sources frequently relies on manual inspections via inspection vehicles, a process that is both inefficient and time-consuming. To tackle this challenge, this paper introduces a novel automated approach for detecting road risk sources, termed the multi-scale lightweight network (MSLN). This method primarily focuses on identifying road surfaces, potholes, and scattered objects. To mitigate the influence of real-world factors such as noise and uneven brightness on test results, pavement images were carefully collected. Initially, the collected images underwent grayscale processing. Subsequently, the median filtering algorithm was employed to filter out noise interference. Furthermore, adaptive histogram equalization techniques were utilized to enhance the visibility of cracks and the road background. Following these preprocessing steps, the MSLN model was deployed for the detection of road risk sources. Addressing the challenges associated with two-stage network models, such as prolonged training and testing times, as well as deployment difficulties, this study adopted the lightweight feature extraction network MobileNetV2. Additionally, transfer learning was incorporated to elevate the model’s training efficiency. Moreover, this paper established a mapping relationship model that transitions from the world coordinate system to the pixel coordinate system. This model enables the calculation of risk source dimensions based on detection outcomes. Experimental results reveal that the MSLN model exhibits a notably faster convergence rate. This enhanced convergence not only boosts training speed but also elevates the precision of risk source detection. Furthermore, the proposed mapping relationship coordinate transformation model proves highly effective in determining the scale of risk sources.

## 1. Introduction

At present, China is in the critical period of the development of intelligent transportation, and the construction of intelligent transportation has an important guiding role in the maintenance and management of basic road facilities. China’s road network stretched to 5.198 million kilometers, marking a growth of 185,600 km over the prior year’s end [1]. Among them, the total mileage of road maintenance is 5.144 million km, accounting for 99.0% of the total mileage of roads [2]. Cracks are one of the main factors affecting the performance of roads.

For a long time, road risk source detection has been an important research topic in academic and engineering circles at home and abroad. From the traditional image processing technology to the current hot deep learning technology, road risk source detection technology is constantly updated and has achieved some excellent research results. Crack detection based on threshold segmentation is the earliest algorithm studied [3]. Oh et al. proposed an iterative threshold segmentation algorithm in 1998 [4], but the threshold needs to be set according to engineering experience. Then, Li et al. proposed an improved OSTU algorithm based on the Hough transform [5]. Next, Sun et al. proposed a detection algorithm based on the adaptive threshold Canny algorithm, which no longer required manual threshold setting [6]. But when the light or noise in the image is not uniform, it still cannot produce a stable effect [7,8,9,10,11,12,13]. Subirats et al. proposed a crack detection method based on continuous wavelet transform, which could effectively detect and segment crack images but still had some noise [14]. Hou et al. proposed research and comparison of road surface detection methods based on data depth enhancement, which mainly adopted a data depth enhancement method integrating WGAN-GP and Poisson migration algorithms to supplement and balance training sample data by generating road pit pictures under different occlusion and different light conditions [15]. Huang et al. described a height mutation recognition method based on 3D data. Compared with the gray threshold method and artificial intelligence method, the three-dimensional detection of pavement potholes by linear structured light scanning was proposed [16]. Cha Y J et al. first applied convolutional neural networks (CNN) to detect cracks in concrete, abandoning the traditional practice of manual feature extraction. CNN can accurately identify cracks in the original images that contain variations in lighting and shadows, significantly improving the level of automation and accuracy of the detection [17]. Cha Y J et al. were also the first to adopt the Faster R-CNN visual detection framework, achieving simultaneous recognition of various structural damages. This framework can accurately detect a variety of damage types, including concrete cracks, different degrees of steel corrosion, bolt corrosion, and steel delamination [18]. Xu et al. used ResNet50, the residual network of cavity convolution optimization, as the backbone neural network to extract features and carry out multi-scale processing and proposed a road scatter detection algorithm based on center mask optimization of the case segmentation model [19]. Tien et al. proposed a crack target recognition method FFA that uses both luminance and morphological features to segment fracture targets, which can effectively improve the accuracy of fracture detection [20]. In recent years, due to the rapid development of deep learning, an increasing number of lightweight road risk source detection methods have emerged. Cha et al. proposed a road surface detection method based on a convolutional neural network, which classifies whether each sub-area contains a crack region by the output value of the model. Gopalakrishnan et al. [21] proposed a road crack detection method based on transfer learning, which used the pre-trained convolutional neural network based on the ImageNet dataset and then fine-tuned it to obtain the final detection model. Chen et al. realized the use of the NB-CNN network for crack detection but could not achieve accurate extraction of crack shape [22]. Carr et al. proposed a methodology utilizing a single stage of the target detection network [23]. RetinaNet for road crack detection uses a neural network to extract feature residual and uses the feature extraction of a multi-scale pyramid network diagram, with a slide on each scale using anchor boxes to generate multiple candidate areas and classifying the candidate region and border position return for detecting cracks [24,25]. Liu et al. proposed PDT-YOLO, an efficient roadside object detection algorithm addressing challenges in multi-scale, occlusion, and model deployment for intelligent roadside perception systems [26]. Yuan et al. proposed a super-resolution reconstruction method for pavement crack images based on an improved generative adversarial network (GAN), addressing the issue of significant disparities in image quality due to variable equipment and illumination conditions during the image-collecting stage. As demonstrated in experiments, the method effectively improves the accuracy of pavement crack detection [27]. Rehana K C et al. proposed the YOLOv5-PD model specifically for detecting common defects in asphalt pavements, such as cracks, patches, and potholes, demonstrating the effectiveness of this model in handling defects of varying scales [28]. Kang D et al. proposed an integrated Faster R-CNN algorithm for detecting crack area, and combined an improved tubularity flow field (TuFF) algorithm to achieve pixel-level crack segmentation. In addition, this method also employed a modified distance transform method (DTM) to accurately measure the thickness and length of cracks [29].

In this paper, a two-stage lightweight network is applied to road hazard source detection. First, a road hazard source dataset based on forward computer vision is constructed, and MobileNetV2 is introduced to modify the categories of the computational model. The bounding box loss functions, Alpha IOU and PolyLoss, are improved, thereby enhancing the main feature extraction network of the model. The MSLN network model is proposed. Experiments show that it can effectively improve the efficiency of model training and detection and increase detection accuracy. Additionally, by utilizing visual coordinate transformation to estimate the scale of hazard sources, the model can estimate the scale of hazard sources, meeting the requirements of road inspection.

## 2. Construction and Augmentation of Road Risk Source Datasets

To obtain the image data of road potholes and sprinkles that can be used for model training, we first obtained the road image of actual road maintenance work. Due to the constraints of acquisition equipment and environment, the obtained image has uneven illumination and many noise points, which leads to low image quality. Therefore, we need to preprocess the image first. The road image is preprocessed by the traditional digital image processing method. A noise removal algorithm is applied to the grayscale image, followed by histogram equalization to boost the contrast of the pits, sprinkling objects, and background, and the first batch of images is obtained. Finally, for the first batch of images, random clipping, rotation, flipping, and other common methods in deep learning are used to augment the images to obtain the final experimental dataset.

### 2.1. Grayscale Images of Risk Sources

**Figure 1:**
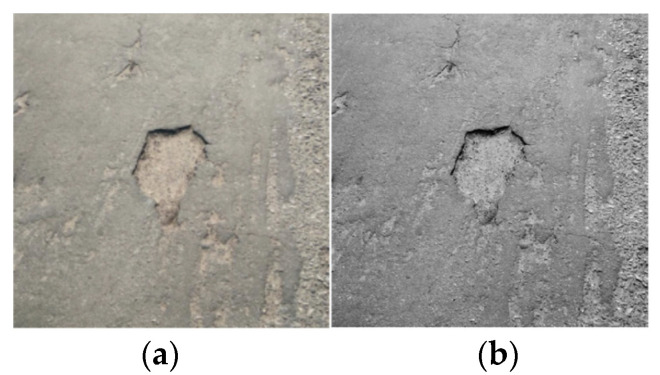
Image grayscale processing: (**a**) the original image; (**b**) road grayscale diagram.

The images collected in road maintenance are RGB three-color channel images, which occupy a large memory. However, in the actual pit and sprinkling recognition and detection work, the pixel difference between the target and the background is large, so the image gray processing does not affect the recognition, and detection work can reduce memory usage and is conducive to the subsequent image noise reduction and enhancement. Therefore, in this paper, the color image is gray processed to obtain a single-channel gray image, mainly taking the pit as an example, as shown in Figure 1.

### 2.2. Crack Image Denoising

In the way of daily maintenance, collected road pavement images are often affected by environmental and human factors, such as a lot of noise, so reducing the noise influence on the crack detection requires a filter algorithm of image noise reduction, which will preserve image details and remove noise as much as possible. This paper mainly uses a Gaussian filtering algorithm to process the image.

**Figure 2:**
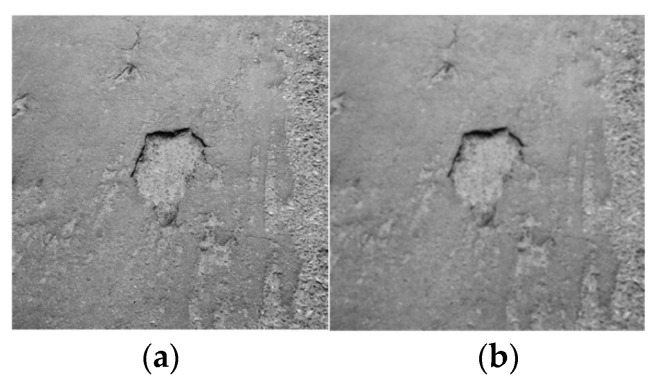
Image processed by Gaussian filtering; (**a**) road grayscale diagram; (**b**) filter processing diagram.

Gaussian filtering is a kind of classic filtering method, which realizes the filter in the same calculation template size of neighborhood pixel gray value of the mean as a filter within the collection after the goal of pixel values is reached; however, in the Gaussian filter, the farther the distance, the smaller the target pixel distance position influence coefficient, thus reducing the after filter in the neighborhood pixel difference caused by the blur effect. Therefore, compared with other filters, Gaussian filter can better retain the detailed information of crack targets [29]. Gaussian noise is a common noise that conforms to the normal distribution model and the probability density function formula such as Equation (1):

(1) $$P(z)=\frac{1}{\sqrt{2\pi }\sigma }e^{-{\left(z-u\right)}^{2}/2\sigma^{2}}$$

where z represents the gray value of the image, u represents the mean value of gray, and σ represents the standard value of gray. The image processed by Gaussian filtering is shown in Figure 2.

### 2.3. Enhancement of the Fracture Image

After image processing with a denoising filtering algorithm, the pavement crack image is relatively smooth. To enhance the difference between the target and background of road cracks and improve the detection and recognition effect, this paper mainly enhances the image by histogram equalization and expands the sample number to enhance the generalization of the model by using common data augmentation methods in deep learning, such as random clipping, rotation, and gamet transformation.

**Figure 3:**
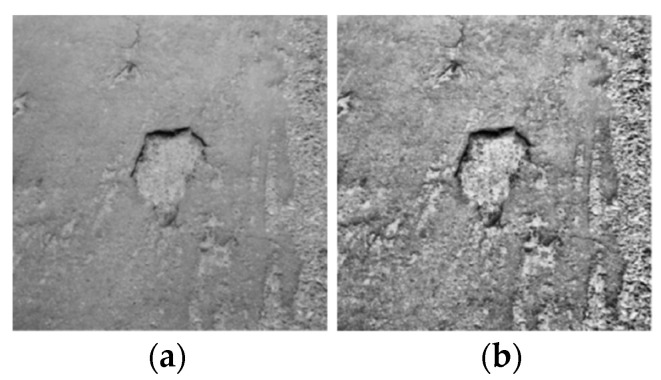
Image processed after equalization: (**a**) filter processing diagram; (**b**) equalization processing diagram.

Histogram equalization is a method to enhance image contrast. Its main idea is to change the histogram distribution of an image into an approximate uniform distribution to enhance the image contrast [30]. A typical histogram equalization formula is presented in Equation (2):

(2) $$S_{i}=T(r_{i})=\sum_{j=0}^{k}\frac{n_{i}}{N}$$

where $S_{i}$ represents the gray value of the output image, $r_{i}$ corresponds to the gray value obtained from the input image, $n_{i}$ is the number of pixels in the gray value $r_{i}$, N is the total number of pixels in the image, and k is the gray level. The sum term indicates the percentage of pixels with a value of i relative to the overall pixel count. The image after equalization is shown in Figure 3.

**Figure 4:**
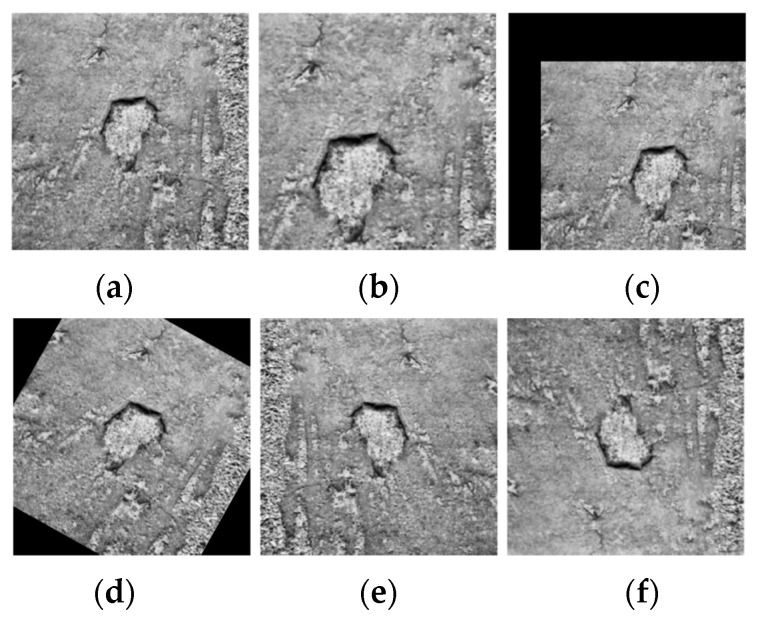
Data augmentation: (**a**) the original image; (**b**) random cutting; (**c**) translation; (**d**) random rotation; (**e**) horizontal reversal; (**f**) vertical flip.

Then, for the images after equalization processing, we used the method of data augmentation to construct the final sample set of road cracks, with a total of 1200 images. The image after data augmentation is shown in Figure 4.

## 3. Recognition Algorithm Based on Lightweight Network

### 3.1. Improve the Trunk Feature Extraction Network

As one of the two-stage target detection networks, Faster RCNN has a long training detection time and high hardware requirements, which is one of its major shortcomings. In the daily road maintenance and inspection scene, the hardware facilities of the terminal equipment often cannot meet the requirements, so it is urgent to develop a lightweight network model to improve this disadvantage and improve the training and detection efficiency of the network model.

**Figure 5:**
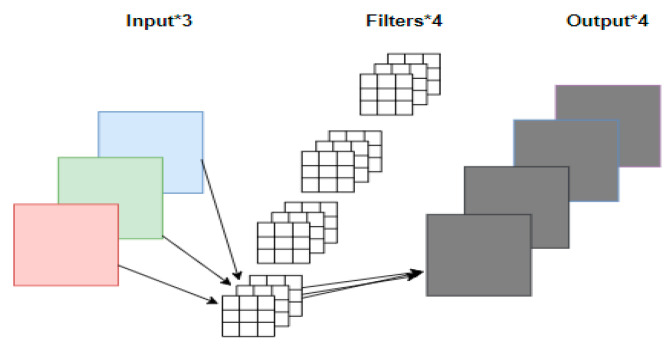
Traditional convolution, * denotes matrix multiplication.

**Figure 6:**
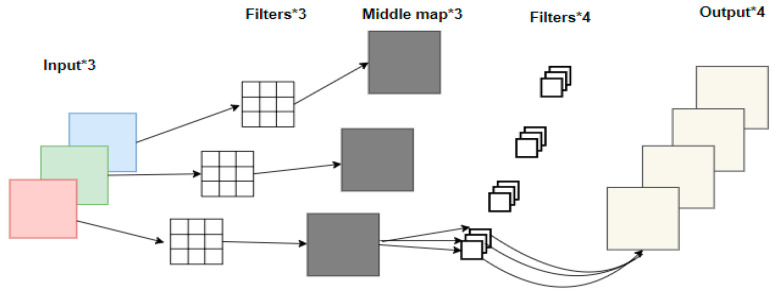
Depth-separable convolution, * denotes matrix multiplication.

MobileNetV2 focuses on lightweight convolutional neural networks in mobile terminals or embedded devices and is proposed to solve the shortcomings of traditional convolutional neural networks, such as large memory requirements and large computation. Depth-detachable convolution in the network can greatly reduce the number of parameters in the operation of our network and improve the operation speed. Traditional convolution and depth-detachable convolution are shown in Figure 5 and Figure 6.

It can be seen from Figure 5 and Figure 6 that in traditional convolution, the convolution kernel channel is equivalent to the input eigenmatrix channel depth. For depth-separable convolution, the DW convolution, with the depth of convolution kernel equal to 1 and the number of convolution kernels as the input eigenmatrix, is first used to obtain the output of the middle layer. The final output is derived by convolving with a 1 × 1 convolution kernel. Assuming that $D_{F}$ denotes the dimensions of the input eigenmatrix, $D_{K}$ denotes the size of the convolution kernel, M denotes the depth of the input eigenmatrix, and N denotes the depth of the output eigenmatrix, the calculation formula of the depthable convolution parameter and ordinary convolution is given Equation (3):

(3) $$\frac{D_{K}\times D_{K}\times M\times D_{F}\times D_{F}+M\times N\times D_{F}\times D_{F}}{D_{K}\times D_{K}\times M\times N\times D_{F}\times D_{F}}=\frac{1}{N}+\frac{1}{D_{K}^{2}}=\frac{1}{N}+\frac{1}{9}$$

When our convolution kernel is 3 times 3, the ordinary convolution is 8 or 9 times bigger than our deep-separable convolution.

**Figure 7:**
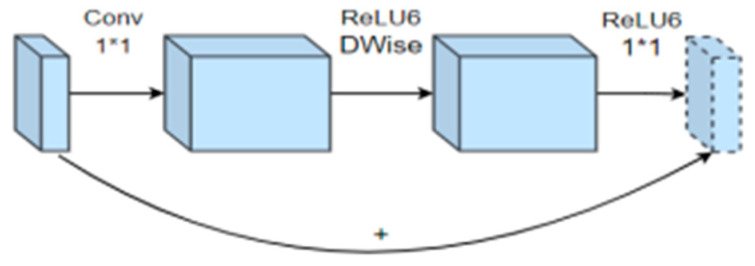
Inverted residual structure, * denotes matrix multiplication.

The backward residual structure in the MobileNetV2 network can increase the feature information extraction of the input image, as shown in Figure 7.

Departing from the conventional residual structure, the traditional approach initially uses a 1 × 1 convolution kernel to compress our input eigenmatrix, that is, to reduce the channel of our input eigenmatrix. And then, it is processed by a 3 × 3 convolution kernel; finally, we use a 1 × 1 convolution kernel to extend its channel. This creates a bottleneck structure that is wide at both ends and narrow in the middle. In contrast, for the inverted residual structure features, the 1 × 1 convolution kernel is first used for dimension raising operation, and the number of channels becomes deeper. Then, the DW convolution operation of the 3 × 3 convolution kernel is used for convolution, and more convolution is extracted for dimension reduction through the 1 × 1 convolution. Therefore, the trunk feature extraction network in Faster R-CNN is replaced with MobileNetV2 to shorten the model training time and improve training efficiency.

### 3.2. Improve Loss Function

The loss function is of great significance in the training of neural networks. In the two-stage target detection network model Faster RCNN, loss in the network is mainly divided into two parts: regional suggestion network loss and Fast RCNN network loss. In the calculation area, the category of the network is suggested, that is, to distinguish the loss between the foreground and the background where binary cross-entropy loss is mainly used, and Smooth L1 loss is mainly used for the positioning loss. In the calculation of the category loss of the Fast RCNN network, cross-entropy loss is mainly used, and Smooth L1 loss is still used for the positioning loss of candidate box coordinates.

To use a more flexible form of the loss function, the PolyLoss function [31] was introduced to replace the cross-entropy loss function in the Fast RCNN network. Designing the loss function as a linear combination of polynomial functions facilitates the adjustment of the importance of different polynomials according to various target tasks and datasets. Its calculation formula is presented in Equation (4):

(4) $$L_{Poly}=\sum_{j=1}^{\infty }\alpha_{j}(1-P_{t}{)}^{j}$$

where $\alpha_{j}$ represents the polynomial coefficient, and $P_{t}$ represents the probability of predicting categories.

Meanwhile, taking into account Smooth L1 losses, its calculation formula is shown in Equation (5):

(5) $${\mathrm{Smooth}\;L}_{1}=\begin{array}{c} 0.5x^{2},|x|<1\\ |x|-0.5,x<-1\;\mathrm{or}\;x>1 \end{array}$$

**Figure 8:**
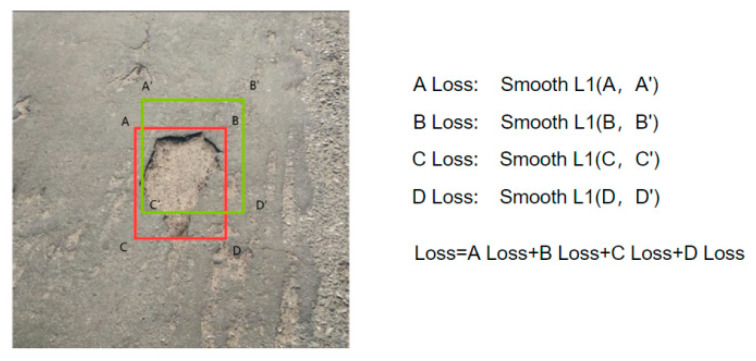
Smooth L1 calculation method.

When calculating the loss of the boundary box, the loss function first independently calculates the loss of four points—A, B, C, and D—and then adds them together, as depicted in Figure 8. In the figure, red represents the ground truth and green represents the prediction box.

**Figure 9:**
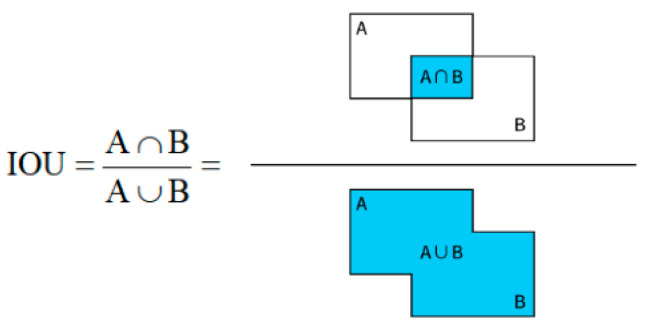
Alpha-IOU calculation method.

This approach presumes the mutual independence of the four points; however, in reality, there exists a correlation between the candidate box’s four coordinates because they all depend on the same target. Therefore, alpha-iOU loss [30] is introduced in this paper to replace Smooth L1 loss of the region suggestion network and Fast RCNN network, respectively, for accurate candidate box regression and target detection. The formula of alpha-iOU loss function is given in Equation (6). The IOU calculation of the candidate box and the ground truth is shown in Figure 9.

(6) $$L_{\alpha -\mathrm{IOU}}=1-{\mathrm{IOU}}^{\alpha }$$

where α represents power operation.

## 4. Dimension Estimation of Disease

In the field of road surface detection, the identification and assessment of the severity of pavement distress are crucial for maintaining road safety and extending the service life of the road. When pavement distress is detected, merely identifying its type and location is insufficient; it is also necessary to calculate its dimensions to more comprehensively evaluate the extent of pavement damage. Dimensional information can assist engineers in determining the severity of the distress, devising appropriate repair schemes, and predicting future maintenance needs. To compute the dimension of pavement distress, it is essential to convert the three-dimensional position information of the distress in the world coordinate system to two-dimensional pixel coordinates in the image coordinate system. This involves a series of complex coordinate system transformation processes requiring the use of camera calibration and pose estimation techniques.

In the domains of image processing and stereo vision, accurately depicting the position and orientation of objects relies on four levels of coordinate systems: the world coordinate system, the camera coordinate system, the image coordinate system, and the pixel coordinate system. These coordinate systems form an interconnected network that translates the position of objects in the real world into pixel values that computers can interpret. The world coordinate system provides a global reference framework, defining the absolute positions of objects within a scene, akin to a coordinate system on a map. The camera coordinate system uses the camera as the reference center, describing the relative position and orientation of objects for the camera. The image coordinate system resides on the imaging plane and is responsible for projecting three-dimensional world coordinates onto a two-dimensional image plane. The pixel coordinate system further converts image coordinates into pixel values that computers can directly process, and it is the most directly relevant coordinate system for computer vision processing. Through this series of coordinate transformations, precise mapping from the real world to digital images can be achieved, providing robust technical support for road distress analysis and object localization.

### 4.1. Camera Parameter Acquisition

**Figure 10:**
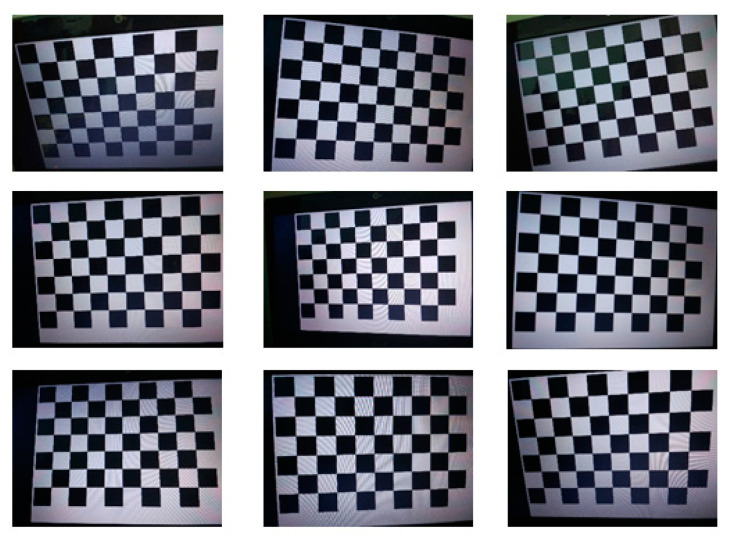
Checkerboard calibration pattern.

According to the Chandraker M calibration method [32], the mapping relationship between the world coordinate system and the camera coordinate system is established. First, Figure 10 shows the chessboard calibration pattern. After obtaining an image of the calibration pattern, an image detection algorithm is applied to detect the pixel coordinates of each corner point. Since the world coordinate system of the calibration pattern is predefined by humans, and the dimension of each square on the calibration board is known, the physical coordinates of each corner point in the world coordinate system can be calculated. Using this information, the camera calibration is performed, yielding the mapping relationship formula Equation (7) between the world coordinate system and the camera coordinate system.

(7) $$\left[\begin{array}{c} x_{c}\\ y_{c}\\ z_{c}\\ 1 \end{array}\right]=\left[\begin{array}{cc} R & T\\ \vec{0} & 1 \end{array}\right]•\left[\begin{array}{c} x_{w}\\ \begin{array}{l} y_{w}\\ z_{w} \end{array}\\ 1 \end{array}\right]$$

where $\left(x_{w},y_{w},z_{w}\right)$ represents the world coordinate system, $\left(x_{c},y_{c},z_{c}\right)$ represents the camera coordinate system, $R^{\left(3\times 3\right)}$ represents the rotation vectors along the x, y, and z axes, and $T^{\left(3\times 1\right)}$ represents the translation vector.

Then, through the conversion from the camera coordinate system to the image coordinate system and from the image coordinate system to the pixel coordinate system, the mapping relationship from the world coordinate system to the pixel coordinate system is obtained, with the formula given as (8).

(8) $$\left[\begin{array}{c} u\\ v\\ 1 \end{array}\right]=\left[\begin{array}{ccc} f_{x} & 0 & u_{0}\\ 0 & f_{y} & v_{0}\\ 0 & 0 & 1 \end{array}\right]•\left[\begin{array}{cc} R & T\\ \vec{0} & 1 \end{array}\right]•\left[\begin{array}{c} X_{w}\\ Y_{w}\\ Z_{w}\\ 1 \end{array}\right]$$

where $Z_{c}$ represents the vertical coordinate in the camera coordinate system, $\left(X_{w},Y_{w},Z_{w}\right)$ denotes the coordinates in the world coordinate system, (u,v) denotes the coordinates in the pixel coordinate system, $\left(u_{0},v_{0}\right)$ represents the coordinates of the camera sensor center in the pixel coordinate system, $\left(f_{x},f_{y}\right)$ represents the camera focal length, R denotes the rotation matrix, and T represents the translation vector.

### 4.2. Camera Pose Estimation

In practical applications, the direct measurement of the rotation matrix R and the translation vector T in Equation (8) presents operational inconvenience and challenges in precision. Therefore, this paper employs camera pose estimation techniques to obtain these parameters.

**Figure 11:**
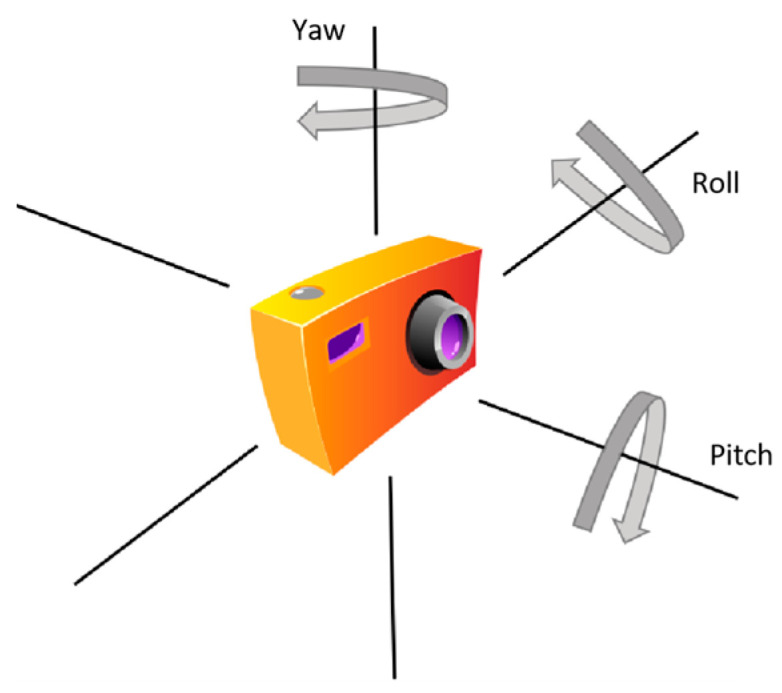
Camera pose illustration.

Specifically, based on the intrinsic and distortion parameters obtained from camera calibration in Section 4.1, we can estimate the camera pose by solving the Perspective-n-Point (PnP) problem. The PnP problem involves calculating the camera’s projection relationship given N feature points in the world coordinate system and their corresponding image points in the image coordinate system, thereby obtaining the pose of the camera or object. This paper employs the P3P algorithm, which is a PnP algorithm that requires only three pairs of matching points to complete the camera pose estimation (Figure 11). Through the P3P algorithm, we can solve for the rotation angles of the camera around the three coordinate axes, i.e., Roll (roll angle), Pitch (pitch angle), and Yaw (yaw angle). These parameters comprehensively describe the camera pose, laying the foundation for subsequent tasks of calculating the disease dimensions.

In the P3P problem, we consider three points—A, B, and C—in space, along with their known corresponding projections a, b, and c on the image plane. The coordinates of A, B, and C in the world coordinate system are known, but we need to solve for their coordinates in the camera coordinate system. According to the derivation, Formula (9) was obtained.

(9) $$(1-u)y^{2}-yx^{2}-\cos(b,c)y+2uxy\cos(a,b)+1=(1-w)x^{2}-wy^{2}-\cos(a,c)x+2wxy\cos(a,b)+1$$

Equation (9) is a binary quadratic equation about x and y. By solving it using the elimination method, a maximum of four solutions may be obtained. Therefore, in addition to the three points, a set of matching points is needed for verification. With this, the coordinates of A, B, and C in the camera coordinate system can be determined using x and y. Hence, the 3D-2D problem transforms into a 3D-3D pose estimation problem.

3D-3D pose estimation pertains to the situation where the three-dimensional coordinates of a point in space are known in two camera coordinate systems. According to the iterative closest point (ICP) algorithm [24], if a series of points in space have their three-dimensional coordinates as $C=c_{1},\cdots c_{n}$ in the first camera coordinate system and as $C^{\prime }=c_{1}^{\prime },\cdots c_{n}^{\prime }$ in the second camera coordinate system, Equation (10) is derived.

(10) $$\forall i,c_{i}=Rc_{i}^{\prime }+T$$

where R, T represent the rotation matrix and the translation vector, respectively.

Then, the linear algebraic solution is applied to Equation (10) to construct the least squares error term as in Equation (11), resulting in the final rotation matrix R and translation matrix T.

(11) $$\underset{R,t}{\min}J=\frac{1}{2}{\sum_{i=1}^{n}\left\|c_{i}-(Rc_{i}^{\prime }+T)\right\|}_{2}^{2}$$

## 5. Experiment

### 5.1. Datasets

In this paper, a highway crack disease dataset was constructed to meet the training requirements of industrial recognition algorithms. The dataset collected part of the pavement disease data from March to September 2022 through the vehicle-mounted front camera and used LabeImg to mark the diseases in each image as scattered objects and potholes, respectively.

The number of dataset divisions and image sizes are shown in Table 1:

### 5.2. Experimental Environment

The experimental setup consists of a Windows operating system running on an Intel^®^ Core™ i5-8265u CPU (Santa Clara, CA, USA), equipped with 16 GB of random-access memory (RAM) and an NVIDIA GeForce RTX 3090 graphics card featuring 24 GB of display memory. The deep learning framework utilized is PyTorch 3.3. For this experiment, a dataset comprising a total of 6000 images was employed, with 5000 images designated as the training set and 1000 images allocated as the test set. To facilitate the differentiation between positive and negative samples during the training phase, this study employs a method to compute the degree of overlap between candidate regions and the ground truth.

The specific screening rules are shown in Table 2:

### 5.3. Analysis of Experimental Results

This paper introduces modifications to the original Faster R-CNN model, enhancing its backbone feature extractor and loss function. Initial training parameters for the enhanced algorithm are listed in Table 3. Table 4 shows the performance of various two-stage object detection models and the proposed model on the road damage dataset.

The experiment results are shown in Table 3. The first group is the baseline Faster RCNN algorithm; the second group modifies the backbone network of Faster RCNN to MobileNetV2 (MF RCNN). The third group modifies the cross-entropy loss of the Fast RCNN part to PolyLoss based on Faster RCNN (PF RCNN), and the fourth group replaces the region of Fast RCNN with smooth L1, namely Alpha IOU (AF1 RCNN). The fifth group replaces the Smooth L1 of the Fast RCNN network part with Alpha IOU (AF2 RCNN). The sixth group is the fusion of the backbone network MobileNetV2 and the loss functions Alpha IOU and PolyLoss, which is the MSLN algorithm proposed in this article.

As can be seen from Table 3, although the MAP of MSLN from RseNet50 to MobileNetV2 was reduced by 0.9%, the training time was reduced by 31.7%, and the model training parameters were reduced by 34.6%, effectively saving training time and improving training efficiency. Based on Faster RCNN, the cross-entropy loss of the Fast RCNN part was modified to PolyLoss, the MAP of PF RCNN reached 61.8%, and the model parameters only increased slightly. After replacing the Smooth L1 loss function of the Faster RCNN with the Alpha IOU loss function, the mAP trained by the model is improved by 0.8% and 0.7%, respectively. The Alpha IOU loss function enhanced model detection accuracy by incorporating inter-boundary box distance metrics. As can be seen from the last line, the detection accuracy of the improved MSLN proposed in this paper reaches 63.1%, and the training time is also greatly shortened compared with the Faster RCNN, which can effectively improve the training accuracy of the model and reduce the trainable parameters of the model.

### 5.4. Experimental Results of Disease Dimension Estimation

By substituting the coordinates of the upper-left and lower-right corners of the MSLN object detection box into Equation (8), we reverse-calculate the specific position of the disease in the world coordinate system. Based on this, the length and width of the disease are represented by Equation (12):

(12) $$\begin{array}{l} w=x_{w2}-x_{w1}\\ h=y_{w2}-y_{w1} \end{array}$$

By capturing images of scattered objects and real pothole diseases in multiple different placement positions, this paper calculates their lengths and widths. To verify the accuracy of the measurements, the calculated dimensions were compared with the actual dimensions of the diseases, and the errors and error ratios in length and width were calculated and are presented in Table 5.

Table 4 illustrates the size errors of scattered objects and potholes in two different placement positions. In the experiment, we tested 10 different scattered objects and potholes. The average length error ratio of scattered objects was 24.2%, and the average width error ratio of potholes was 32.2%. The overall results showed that the errors of scattered objects were generally smaller. Furthermore, as can be roughly seen from the pattern in Table 4, larger objects or diseases generally have smaller errors, such as Disease 3, Disease 5, and Disease 6. Since road inspection mainly aims to identify significant risk sources, this work meets the actual needs of highway inspection and is significant for the detection and identification of road risk sources.

## 6. Results and Discussion

**Figure 12:**
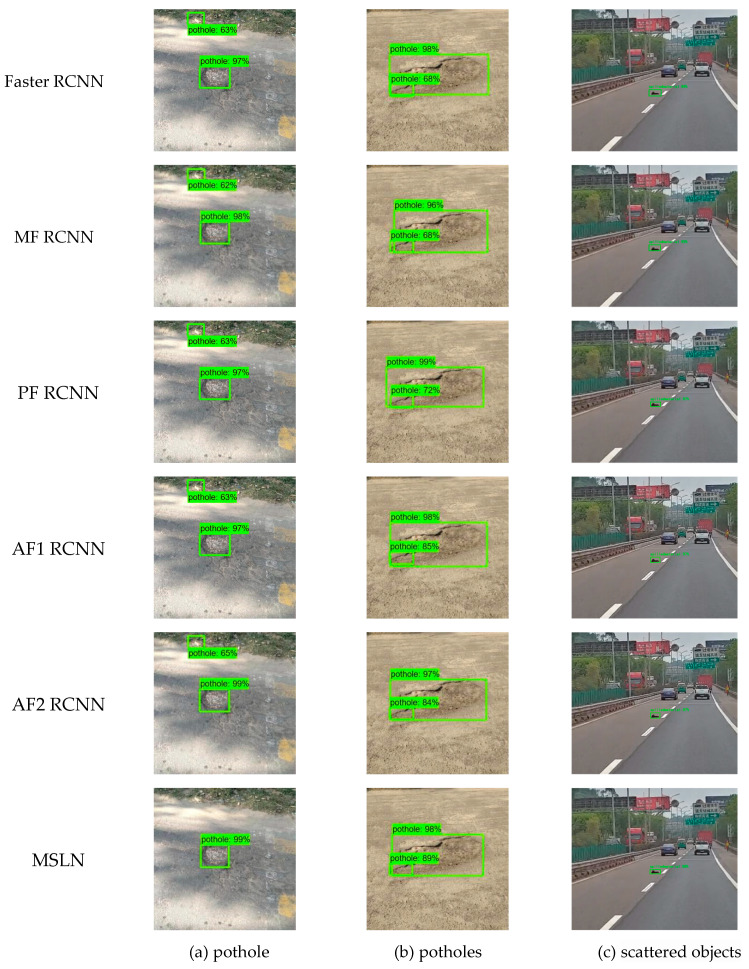
Comparison of experimental results.

Three images in the test set were randomly selected for testing of different models, and the results are shown in Figure 12.

As can be seen from Figure 12a, both basic Faster RCNN and AF RCNN, which individually modified the loss function or the trunk feature extraction network, identify the white paper in the upper-left corner of the picture as a pit. However, the MSLN proposed in this paper, on the one hand, does not have false recognition and reduces the false positive rate. On the other hand, it also improves the recognition rate of pits. It can be seen in Figure 12b that the basic Faster RCNN model can identify overlapping pit and groove diseases, but the detection accuracy of small targets in the overlapping part is low. After the Smooth L1 loss function in the Faster RCNN is modified, the detection accuracy of small targets in the overlapping part can be significantly improved. Finally, the small target detection accuracy of the overlapping part of MSLN proposed in this paper reaches 89%. Figure 12c illustrates that the Faster R-CNN model tailored for scattered object detection achieves notable recognition performance; however, by further enhancing the feature extraction network and the loss function, our proposed MSLN managed to elevate the detection accuracy for scattered objects even further. To sum up, the MSLN proposed in this paper can reduce the false positive rate of detected objects on the one hand and improve the detection accuracy of objects on the other hand, thus improving the detection efficiency of the algorithm.

**Figure 13:**
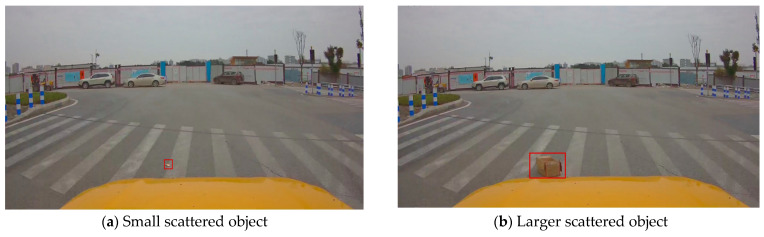
Scattered object dimension test.

Figure 13 presents the dimension measurement results of two different-volume scattered objects. Figure 13a reflects the measurement of scattered object disease 1 listed in Table 4. Figure 13b depicts another scattered object. In Figure 13b, it can be observed that for scattered objects with a certain height, the dimension calculation error is relatively large. This is because the target detection box typically only provides two-dimensional position information of the object in the image, which limits the accuracy of the conversion from the image to the three-dimensional space. When a scattered object has a certain height, the two-dimensional information is converted into a three-dimensional position in the world coordinate system. The calculation error in the Z-axis is particularly significant, which directly affects the accuracy of the dimension measurement.

## 7. Conclusions

This paper presents a novel MSLN approach for the automated detection of road risk sources, including road surfaces, potholes, and scattered objects. The proposed method addresses the inefficiencies of traditional manual inspection methods by leveraging advancements in deep learning and computer vision techniques. The MSLN model integrates several key innovations to improve the detection efficiency and accuracy. First, MobileNetV2, a lightweight feature extraction network, was adopted to replace the conventional backbone network, significantly reducing the training time and hardware requirements. Second, the loss functions were optimized by introducing PolyLoss and Alpha IoU loss, improving the model’s ability to detect objects accurately. Furthermore, a mapping relationship model was established to convert the detected objects from the pixel coordinate system to the world coordinate system, enabling the estimation of the dimensions of road risk sources. Experimental results demonstrate the effectiveness of the proposed MSLN model. Compared with the baseline Faster R-CNN model, the MSLN achieved a notable improvement in both training speed and detection accuracy, with a mean average precision (MAP) of 63.1% and a reduced training time of 31.7%. The mapping relationship model also showed promising results in estimating the dimensions of scattered objects and potholes.

The method proposed in this paper has demonstrated its value over an application journey of more than 20,000 km, but at the same time, we have also discovered its limitations in the actual environment. Especially under conditions of insufficient ambient light and backlight, the recognition was significantly affected. Future research will focus on enhancing the MSLN model in several directions. First, backbone fusion networks that combine lightweight and powerful networks will be explored to improve detection accuracy without sacrificing efficiency. Second, we will develop recognition algorithms for road conditions under low-light and backlight conditions to enhance their identification accuracy. Third, depth information from depth cameras or stereo vision will be incorporated to enhance dimension estimation, particularly for objects with height. Real-time detection performance and ease of deployment will be optimized through wider testing and efficient data processing pipelines. Multimodal data fusion, combining image data with LiDAR and radar, will be investigated to improve robustness in complex environments. Finally, the model’s generalizability to other road infrastructures, such as bridges and tunnels, will be assessed to broaden its application scope. These efforts aim to refine and extend the MSLN model for widespread adoption in intelligent transportation systems.

## Figures and Tables

**Table 1:** Dataset division and image size.

Dataset	Number	Image Size
Training set	5000	1080 × 720
Validation set	1000	1080 × 720
Test set	1000	1080 × 720

**Table 2:** Filtering rules.

IOU	Sample Classification	Tag
>0.7	Positive samples	1
[0.3, 0.7]	Negative samples	−1
<0.3	Abandon the sample	0

**Table 3:** Model initialization parameters.

The Size of the Batch	Basis Vector	Vector Attenuation Way	The Weight Damping Item	Learning Rate Parameter	Training Number of Rounds
12	0.01	Step	0.0005	0.1	200

**Table 4:** Details of MSLN and Faster RCNN experimental results.

Algorithm	Backbone	Loss Function	Training Time/min	MAP	Model Parameters	FPS
Faster RCNN	RseNet50	L1 Loss + cross-entropy loss	148.2	61.2%	82385006	24
MF RCNN	MobileNetV2	L1 Loss + cross-entropy loss	105.3	61.1%	61352281	27
PF RCNN	RseNet50	PolyLoss	135.1	61.8%	62746534	27
AF1 RCNN	RseNet50	Alpha IOU	135	62.0%	67894657	26
AF2 RCNN	RseNet50	Alpha IOU	134.9	61.9%	68456536	26
MSLN(ours)	MobileNetV2	PolyLoss + Alpha IOU	**101** **.2**	**63.1%**	**53845968**	**32**

**Table 5:** Errors in disease dimension calculation.

Type	Number	True Length (cm)	True Width (cm)	Estimated Length (cm)	Estimated Width (cm)	Length Error	Width Error	Average Error
Scattered objects	Disease 1 (pos1)	11	11	16	13	5	2	34.1%
	Disease 1 (pos2)	11	11	7	15	4	4	
	Disease 2 (pos1)	18	7	20	13	2	5	39.1%
	Disease 2 (pos2)	18	7	21	12	3	4	
	Disease 3 (pos1)	**41**	**15**	30	15	9	0	**9.6%**
	Disease 3 (pos2)	**41**	**15**	37	16	4	1	
	Disease 4 (pos1)	7	18	10	21	3	3	25.4%
	Disease 4 (pos2)	7	18	8	23	1	5	
	Disease 5 (pos1)	**53**	**30**	60	35	7	5	**12.7%**
	Disease 5 (pos2)	**53**	**30**	59	26	4	4	
Pothole	Disease 6 (pos1)	**41**	**41**	57	37	16	4	**20.7%**
	Disease 6 (pos2)	**41**	**41**	27	14	14	0	
	Disease 7 (pos1)	19	6	25	9	6	3	41.0%
	Disease 7 (pos2)	19	6	22	10	3	4	
	Disease 8 (pos1)	18	7	13	10	5	3	25.4%
	Disease 8 (pos2)	18	7	15	6	3	1	
	Disease 9 (pos1)	12	6	10	9	2	3	39.6%
	Disease 9 (pos2)	12	6	11	11	1	5	
	Disease 10 (pos1)	8	4	11	5	3	1	34.3%
	Disease 10 (pos2)	8	4	12	3	4	1	

## Data Availability

Due to privacy restrictions, some data are not publicly available but can be made available from the authors upon reasonable request and with permission from the China Merchants Chongqing Road Engineering Inspection Center Co., Ltd.
